# Genetic diversity and relatedness of Candida albicans isolates from humans and animals in Iran: implications for infection control

**DOI:** 10.3205/dgkh000654

**Published:** 2026-06-05

**Authors:** Esmaeil Hayati, Mansour Bayat, Kumars Amini, Mohammad Hossein Yadegari

**Affiliations:** 1 Department of Veterinary Pathobiology, SR.C., Islamic Azad University, Tehran, Iran; 2 Department of Microbiology, Sav.C., Islamic Azad University, Saveh, Iran; 3 Department of Medical Mycology, Faculty of Medical Sciences, Tarbiat Modares University, Tehran, Iran

**Keywords:** Candida albicans, drug susceptibility, RAPD-PCR, human and animal infection

## Abstract

**Introduction:**

*Candida* species are known to be a major cause of fungal infections in both humans and animals worldwide. Understanding the local epidemiology and genetic diversity of *Candida albicans* is essential for effective control and prevention of infections on a global scale. In this study, we aimed to evaluate the genetic relatedness between human and animal isolates of *Candida albicans* in Iran.

**Methods:**

Thirty-six isolates of *C. albicans* were obtained from both human and animal sources and were subjected to random amplified polymorphic DNA-polymerase chain reaction (RAPD-PCR) using three primers (OPE-04, OPE-18, and OPA-18). Identification, antifungal susceptibility testing, and virulence profiling were carried out using standardized methods.

**Results:**

Our analysis revealed a significant genetic separation between human and animal isolates of *C. albicans*, with distinct genotypes identified in each group. Some degree of genotypic relatedness was observed, but with varying levels of separation. The highest genetic similarity (84%) was observed between *C. albicans* isolates from cow feces and human high vaginal swabs, indicating genetic relatedness but not clonal identity. No clonal relationship was detected among the isolates.

**Conclusion:**

Although this study represents a preliminary investigation, our findings suggest a high discriminatory power of the three primers used in RAPD-PCR for typing Candida isolates. While RAPD-PCR effectively identified genetic diversity, its reproducibility and resolution remain limited, necessitating further validation using more precise molecular methods. The lack of clonal relationship and the observed genetic diversity highlight the potential for transfer of virulent and resistant strains between humans and animals. Further studies with larger sample sizes are needed to validate these findings and to assess the implications of close contact between humans and animals in the transmission of *Candida* infections.

## Zusammenfassung

**Einleitung:**

Candida-Arten sind weltweit als Hauptursache für Pilzinfektionen bei Mensch und Tier bekannt. Das Verständnis der lokalen Epidemiologie und der genetischen Vielfalt von *Candida (C.) albicans* ist entscheidend für die wirksame Kontrolle und Prävention von Infektionen auf globaler Ebene. In dieser Studie haben wir die genetische Verwandtschaft zwischen humanen und tierischen *C. albicans*-Isolaten im Iran untersucht.

**Methode:**

36 *C. albicans*-Isolate wurden aus humanen und tierischen Quellen gewonnen und mittels Random Amplified Polymorphic DNA-Polymerase-Kettenreaktion (RAPD-PCR) mit drei Primern (OPE-04, OPE-18 und OPA-18) analysiert. Die Identifikation, die Prüfung der Empfindlichkeit gegenüber Antimykotika und das Virulenzprofil wurden mit standardisierten Methoden durchgeführt.

**Ergebnisse:**

Unsere Analyse zeigte eine signifikante genetische Trennung zwischen humanen und tierischen *C. albicans*-Isolaten, wobei in jeder Gruppe unterschiedliche Genotypen identifiziert wurden. Eine gewisse genetische Verwandtschaft wurde festgestellt, jedoch mit variierenden Abstufungen der Trennung. Die höchste genetische Ähnlichkeit (84%) wurde zwischen *C. albicans*-Isolaten aus Kuhkot und humanen hochvaginalen Abstrichen beobachtet, was auf eine genetische Verwandtschaft, aber keine klonale Identität hinweist. Eine klonale Beziehung zwischen den Isolaten konnte nicht nachgewiesen werden.

**Schlussfolgerung:**

Obwohl diese Studie eine vorläufige Untersuchung darstellt, deuten unsere Ergebnisse auf eine hohe Diskriminierungskraft der drei in der RAPD-PCR verwendeten Primer zur Typisierung von Candida-Isolaten hin. Während RAPD-PCR genetische Vielfalt effektiv identifizierte, bleiben ihre Reproduzierbarkeit und Auflösung begrenzt, so dass eine weitere Validierung mit präziseren molekularen Methoden erforderlich ist. Das Fehlen einer klonalen Beziehung und die beobachtete genetische Vielfalt unterstreichen das Potenzial für die Übertragung virulenter und resistenter Stämme zwischen Mensch und Tier. Zukünftige Studien mit größeren Stichproben sind erforderlich, um diese Ergebnisse zu validieren und die Auswirkungen des engen Kontakts zwischen Mensch und Tiern auf die Übertragung von *Candida*-Infektionen zu bewerten.

## Introduction

*Candida* spp. are commensal organisms in both humans and animals [1] and have been isolated from various hosts, including rheas, dogs, horses, goats, and sheep [2]. Candidiasis, caused by *Candida* spp., represents a significant health concern in both human and animal populations, presenting a substantial global public health challenge [3]. Animals can serve as reservoirs of Candida infections for humans or other animals, whether of the same or different species [4]. Human Candida infections have significantly increased in recent years, posing additional challenges to healthcare systems [5].

*Candida (C.) albicans* predominates as the most commonly identified pathogen within the Candida genus, responsible for the majority of infections [6]. Nevertheless, recent research has highlighted a rise in infections attributed to non-albicans *Candida* spp. Despite the administration of antifungal agents, disseminated candidiasis is associated with a high mortality rate (approximately 40-60%). Factors such as inadequate diagnosis, suboptimal disease management, and the compromised health status of patients further contribute to the elevated mortality rates [7].

Major risk factors for Candida infections encompass antibiotic usage, immunosuppressive therapies, and the presence of intravenous catheters. Age, nutritional status, and certain underlying medical conditions can also predispose individuals to candidiasis [8]. Furthermore, extracellular hydrolytic enzymes represent prominent virulence factors in *C. albicans* [9].

Research on candidiasis in animals and its potential connection to human infections remains limited. Similarly, information regarding the role of animals as reservoirs for pathogenic and drug-resistant Candida species is scarce [10]. Data concerning the identities and origins of infecting strains are lacking. Understanding the genetic relatedness between human and animal Candida isolates and the potential for transmission from animals to humans is insufficiently studied, particularly in our designated research area [11].

To address these knowledge gaps and elucidate the molecular epidemiology of *Candida* spp. in Iran, molecular typing investigations are imperative. Such studies hold significance from an epidemiological standpoint and are critical for enhancing global strategies for the management and control of candidiasis [12].

The use of RAPD-PCR in this study is significant as it is a rapid and cost-effective method for genotyping *Candida* spp. without requiring species-specific sequences [13]. By applying short arbitrary primers that anneal to multiple genomic sites, RAPD-PCR can detect strain polymorphisms and provide insights into the genetic relatedness between human and animal *C. albicans* spp. [14].

The findings of this preliminary study can shed light on the potential for transmission of *C. albicans* between humans and animals [15]. Understanding the genetic relatedness of Candida isolates from different sources is crucial for identifying possible reservoirs of infection and routes of transmission. This information can inform control measures and help prevent the spread of Candida infections in both human and animal populations.

Further research using molecular typing techniques like RAPD-PCR can expand our knowledge of the epidemiology of Candida infections, enhance surveillance efforts, and contribute to the development of effective strategies for managing and preventing candidiasis. By investigating the genetic diversity and relatedness of *Candida* spp. in both human and animal populations, we can gain a more comprehensive understanding of the dynamics of Candida infections and improve public health outcomes.

## Methods

### Origin of samples, cultivation and phenotypic identification 

A total of 30 clinical samples, comprising 15 *C. krusei* and 15 *C. parapsilosis* samples, were obtained from the Imam Khomeini Hospital laboratory. Additionally, 30 animal samples, including 15 *C. krusei* and 15 *C. parapsilosis* samples, were received from veterinarians in dairy farms around Tehran. All the collected samples were inoculated onto Sabouraud dextrose agar and incubated. After the colonies grew, a loop was used to transfer some of them to chromogenic agar, where they were incubated at 35°C for 48 hours. The color of the colonies grown on chromogenic agar was examined for the identification of the isolated strains. To perform this test, colonies grown in Sabouraud dextrose agar that had been incubated for 24 to 48 hours and fresh serum were used. A small amount of the yeast colonies were taken with an inoculating loop and suspended in fresh serum to prepare a suspension. The suspension was kept at 37°C for 2 to 3 hours. A drop of the suspension was taken with an inoculating loop and placed on a sterile slide, then covered with a coverslip. The presence or absence of hyphae (short and early hyphae directly produced from the swollen cells) was examined using a bright-field microscope.

### Genomic DNA extraction 

Genomic DNA extraction was carried out as follows: The *Candida* strains were cultured on Sabouraud Dextrose Agar (SDA; Difco) for 48 hours at 28°C to establish a pre-inoculum. Subsequently, 3 x 10^8^ CFU/ml were inoculated into 50 ml of SDA and incubated for an additional 48 hours at 28°C. The resulting cellular biomasses were harvested by centrifugation at 10,000 x g, followed by resuspension in 5 ml of 0.1 M sodium citrate/1.1 M sorbitol buffer (pH 5.5) supplemented with 5 mg/ml of glucanase enzyme. This suspension was then subjected to a 3-hour incubation at 33°C in a shaking water bath to generate protoplasts. The protoplasts obtained were then transferred to 5 ml of lysing buffer (composed of 0.04 M Tris HCl, pH 8.0; 0.20 M NaCl, SDS, and 0.01 M Na2 EDTA), washed thrice with 5 ml of phenol-chloroform, and subsequently precipitated using absolute ethanol and 0.3M NaCl. The resulting precipitate was centrifuged, washed twice with 70% ethanol, dried, and finally resuspended in 100 ml of 0.10 mM Tris HCl (pH 7.5). DNA aliquots were then diluted to a concentration of 50 ng/ml in preparation for the RAPD reaction [12].

### Multiplex PCR 

The DNA extracted from the samples was used for molecular identification of antifungal resistance genes. Multiplex PCR was utilized for simultaneous identification of genes, aiming for time and cost efficiency. The primers [13], contents, and reaction programs used are presented in Table 1 (Tab. 1).

### RAPD-PCR 

RAPD profiles were generated using a 30-µl reaction volume comprising a 1X buffer (Promega), 0.2 mM each of dATP, dGTP, dCTP, and dTTP (Promega), 50 ng of genomic DNA, 2 mM of MgCl_2_ (Promega), 160 nM of primer (Operon), and 1 unit of Taq thermostable DNA polymerase (Promega). The amplification protocol entailed 35 cycles, with denaturation at 95°C for 45 seconds, primer annealing at 36°C for 2 minutes, and extension at 72°C for 2 minutes. Initial denaturation lasted 5 minutes in the first cycle, while the final extension was set to 7 minutes in the last cycle.

Decamer primers from the OPERON kit (OPA 01, 02, 03, 07, 08, 09, and 10) along with arbitrary primers SOY, RP1-4, RP-2, and RP4-28 were utilized for the reactions. Additionally, amplification was conducted using ribosomal primers NS1, NS2, ITS1, and ITS-424, with the melting temperature adjusted to 45°C. The specific primer sequences [14] can be found in Table 2 (Tab. 2).

Fingerprints were generated through electrophoresis of 10-µl aliquots of the reaction on 1.5% agarose gels, which were electrophoresed in TBE (0.45 M Tris borate, 0.001 M EDTA) buffer at 120 V for 90 minutes. Subsequently, the gels were stained with 1 µg/ml ethidium bromide and visualized under UV light using a Polaroid camera (Model DS-34) equipped with black and white film (Type 667, Polaroid Corp.). In each experimental run, the base pair sizes were determined by referencing size markers present in every gel, such as DNA lambda/Hind III or 100-bp ladder from Gibco-BRL.

### Multiple-locus variable-number tandem-repeat analysis (MLVA) 

For each primers (Table 3 (Tab. 3)) set [8], polymerase chain reactions (PCRs) were carried out in a final volume of 20 µl, comprising 1 µl of DNA, deoxynucleoside triphosphates at a concentration of 200 µM each, a forward primer, a 5'-dye-labeled reverse primer at 0.25 µM each, and 1 unit of Taq DNA polymerase (Promega, Madison, WI). The thermal cycling conditions consisted of an initial denaturation step at 95°C for 5 minutes, followed by 35 cycles of denaturation at 95°C for 30 seconds, annealing at 52°C for 30 seconds, extension at 72°C for 45 seconds, and a final extension step at 72°C for 10 minutes. Subsequently, amplicons from each PCR reaction for a given isolate were combined prior to multiplex fragment analysis using a Ceq 8000 Genetic Analyzer (Beckman Coulter, Fullerton, CA, USA). Strain IHEM 9670 served as a control in all experiments. To facilitate interlaboratory comparisons of multiple-locus variable-number tandem repeat analysis (MLVA) results, the amplicon sizes were reported as the exact sequence length (determined through sequencing of representative alleles at each locus), given the dependency of allele sizing on dyes and the specific analyzer utilized for electrophoresis. The method's reproducibility and stability were evaluated following established protocols. Primer specificity was verified by examining 11 non-*C. glabrata* reference strains, including *C. albicans* IHEM 9559, *C. dubliniensis* IHEM 14280, *C. tropicalis* CBS 1920, *C. parapsilosis* IHEM 9557, and *C. krusei* IHEM 9560.

The discriminatory power (D) of Multiple Loci VNTR Analysis (MLVA) was determined using the formula proposed by Hunter and Gaston [15]. To classify the genetically unrelated isolates based on their distance, hierarchical clustering analysis was conducted using R software along with the pvclust package. The potential associations between genotypes and the origins of isolates (clinical data, sex, ward, and anatomical sites) were examined through hierarchical clustering analysis combined with canonical discriminant analysis using Tanagra software.

### Dendrogram plot 

The dendrogram construction and analysis of bands obtained from electrophoresis of the study samples were performed using NTSYS version 2.02e software. Initially, scoring of the bands resulting from the marker electrophoresis was done as quantitative data of zero (0) and one (1) (presence or absence of a band). Subsequently, genetic similarity based on the zero and one data was calculated using Jaccard and Dice coefficients and simple matching. To assess the efficiency of the RAPD-PCR method through cluster analysis based on similarity coefficients, the coefficient of cophenetic correlation was utilized. For strain grouping, cluster analysis using the UPGMA method based on the similarity coefficient with the highest cophenetic correlation coefficient was employed.

A total of 36 *C. albicans* isolates were obtained. However, in this study, only a representative sample of 15 *C. albicans* isolates was genotyped due to cost constraints. It was hypothesized that similarities in enzymatic and antifungal susceptibility profiles between human and animal *C. albicans* isolates could have a significant impact on their genetic similarity. Therefore, animal and human *C. albicans* isolates with similar enzymatic and antifungal resistance profiles were specifically selected for RAPD-PCR analysis. Additionally, the isolates were chosen based on their robust enzymatic activities and resistance to azole antifungal drugs tested in this study. All ethical guidelines regarding experiments involving humans and animals were strictly followed, and ethical clearance for the study was obtained from Imam Khomeini Hospital Ethics Committee, Tehran, Iran.

### Identification, enzymatic activity, and antifungal susceptibility testing of C. albicans 

The specimens were cultured on Sabouraud dextrose agar (SDA) supplemented with 1% chloramphenicol (0.05g/L) and subsequently incubated at 37°C for 24-48 hours. The identification of *C. albicans* was determined based on colony morphology, germ tube test, and growth characteristics on CHROMagar *Candida* [16].

The virulence profile, including enzymatic activity for proteinase, phospholipase, lipase, and hemolysin, was assessed using plate methods, and precipitation zone (Pz) values were determined following the protocol described by Price et al. [17].

Antifungal susceptibility testing was performed using the disk diffusion method. The inhibition zone diameter (IZD) was measured and interpreted according to the Clinical and Laboratory Standards Institute (CLSI) approved protocol, CLSI document M44-A2. Additionally, the minimum inhibitory concentration (MIC) was determined using the broth microdilution method. MIC values for fluconazole and itraconazole were compared to the CLSI interpretative guidelines on susceptibility testing.

### DNA typing for genotypic relatedness using random amplified polymorphic DNA-polymerase chain reaction (RAPD-PCR) 

Genomic DNA was extracted using the QuickDNA Fungal/Bacterial Miniprep Kit (Zymo Research, Irvine, CA, USA), following the manufacturer’s protocol. The extraction process was carried out as per the manufacturer's instructions. The DNA samples were then amplified using oligonucleotide primers. The amplification reactions took place in a DNA thermal cycler from Bio-Rad in Foster City, California, USA. The DNA sequences of the primers used for genotyping were as follows: OPE-18 5'-GGACTGCAGA-3', OPA-03 5'-AGTCAGCCAC-3', and P4 5'-AAGAGCCCGT-3' [18]. 

The amplification reaction was performed in a final volume of 25 µL, containing 1 µL of genomic DNA, 1.25 units of Taq DNA polymerase, 0.3 mM of each four deoxynucleoside triphosphate, 1.5 mM of MgCl2, 0.4 µM of each individual primer, and 2.5 µL of 10x PCR buffer. The reaction conditions included an initial denaturation at 94 °C for 4 min, followed by 32 cycles of 94 °C for 1 min, 42 °C for 30 s, and 72 °C for 90 s, with a final extension at 72 °C for 10 min.

The amplified products were run on a 1.5% agarose gel in tris-borate-EDTA (TBE) buffer for 60 min at 110 V and compared with Gene Ruler 100 bp or 1 kb ladder from Thermo Scientific in London, UK. The gel was stained with ethidium bromide and photographed using ultraviolet photography. Polymorphism was detected based on the presence or absence of bands of sizes and intensity. All PCR reactions were performed in triplicate to ensure reproducibility. RAPD-PCR DNA banding patterns were used to create DNA fingerprints, and dendrograms were constructed using UPGMA method with arithmetic means in the NCSS statistical package.

Isolates with 100% similarity were considered identical, while those with values between 80 to 99% were deemed related but not identical, possibly indicating microevolution of a single strain. Isolates with values below 80% were considered unrelated. To ensure reliable results, it is recommended to use multiple primers and conduct a comparative analysis. Therefore, the dendrogram was constructed using a combination of the three primers [19].

## Results

A total of 30 clinical samples, comprising 15 *C. krusei* and 15* C. parapsilosis* samples, were obtained from the Imam Khomeini Hospital laboratory. Additionally, 30 animal samples, including 15 *C. *krusei and 15 *C. parapsilosis* samples, were received from veterinarians in dairy farms around Tehran.

**Figure 1:**
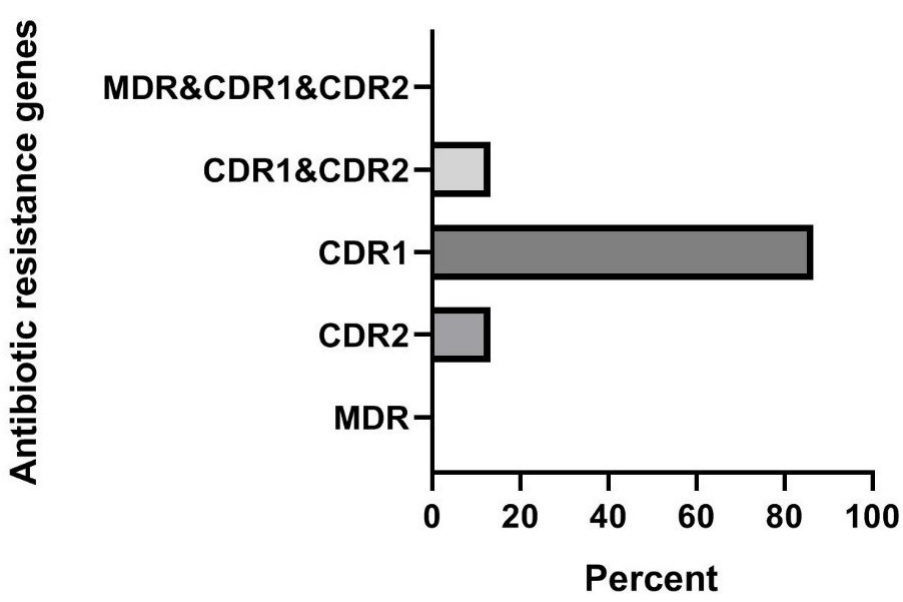
Investigation of the frequency of antibiotic resistance genes in clinical samples of *C. krusei*

**Figure 2:**
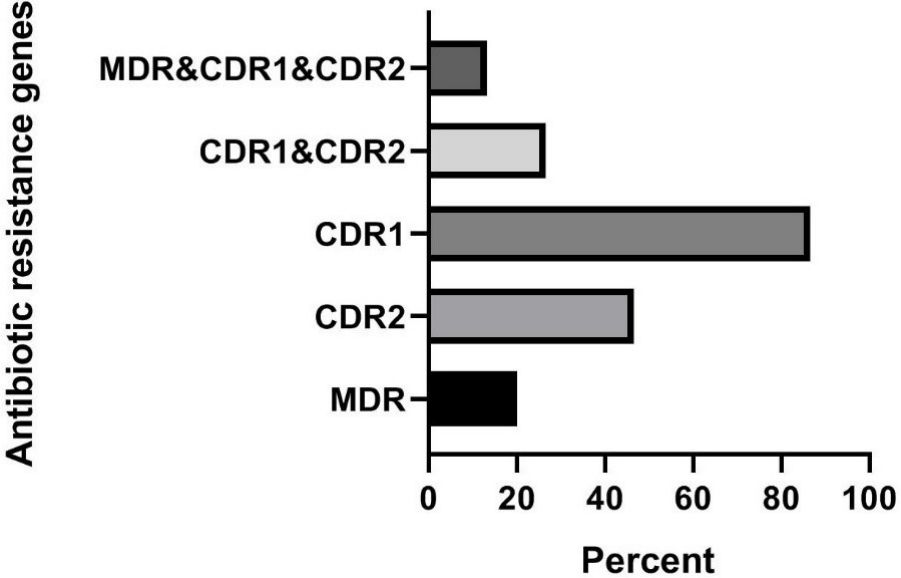
Investigation of the frequency of antibiotic resistance genes in animal samples of *C. krusei*

Based on the results presented in Figure 1 (Fig. 1), in clinical isolates of *C. krusei*, the frequencies of the genes were as follows: MDR1 gene 0% (0 out of 15 samples), CDR2 gene 13.3% (2 out of 15 samples), CDR1 gene 86.7% (13 out of 15 samples), the simultaneous presence of the CDR1 and CDR2 genes 13.3 % (2 out of 15 samples), and the frequency of the simultaneous presence of the three genes MDR1, CDR2, and CDR1 was also 0% (0 out of 15 samples). In animal isolates of *C. krusei*, the frequencies of the genes were: MDR1 gene 20% (3 out of 15 samples), CDR2 gene 46.7% (7 out of 15 samples), CDR1 gene 86.7 % (13 out of 15 samples), simultaneous presence of the CDR1 and CDR2 genes 26.7% (4 out of 15 samples), and the frequency of the simultaneous presence of the three genes MDR1, CDR2, and CDR1 was also 13.3% (2 out of 15 samples) (Figure 2 (Fig. 2)).

In clinical isolates of *C. parapsilosis*, the frequencies of the genes were: MDR1 gene 40% (6 out of 15 samples), CDR2 gene 40% (6 out of 15 samples), CDR1 gene 80% (12 out of 15 samples), simultaneous presence of CDR1 and CDR2 genes 6.7% (1 out of 15 samples), and the frequency of the simultaneous presence of the three genes MDR1, CDR1, and CDR2 was also 33.3% (5 out of 15 samples) [1], [2]. In animal isolates of *C. parapsilosis*, the frequencies of the genes were: MDR1 gene 40% (6 out of 15 samples), CDR2 gene 66.7% (10 out of 15 samples), CDR1 gene 86.7% (13 out of 15 samples), simultaneous presence of the CDR1 and CDR2 genes 33.3% (5 out of 15 samples), and the frequency of the simultaneous presence of the three genes MDR1, CDR1, and CDR2 was also 40% (6 out of 15 samples).

**Figure 3:**
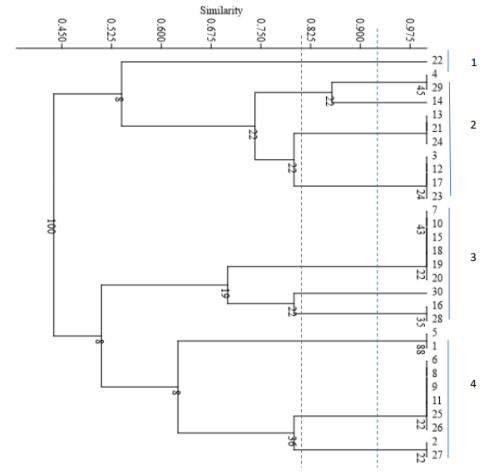
Phylogenetic tree based on the analysis of microsatellite results of *C. krusei* samples

**Figure 4:**
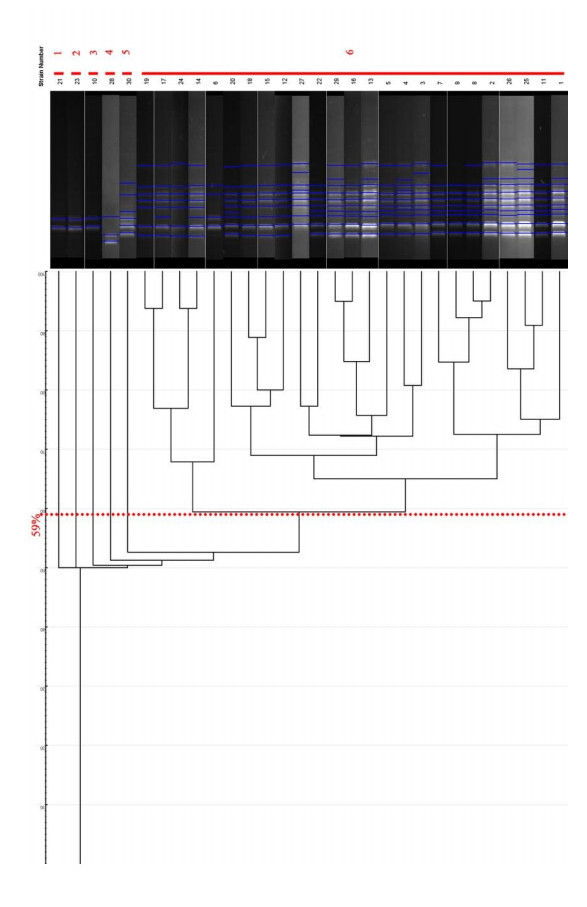
The dendrogram is drawn based on RAPD-PCR for *C. krusei*

All strains of *C. krusei* examined at a similarity level of 59% were divided into 6 distinct groups, such that 1 isolate was placed in the first, second, third, fourth, and fifth groups, and 25 isolates were placed in the sixth group. The results of this part of the study are presented Figure 3 (Fig. 3). The Simpson's coefficient calculated for RAPD-PCR was 31.0, indicating the low discriminatory power of this method for genotyping and polymorphism analysis of *C. krusei* (Figure 4 (Fig. 4)).

**Figure 5:**
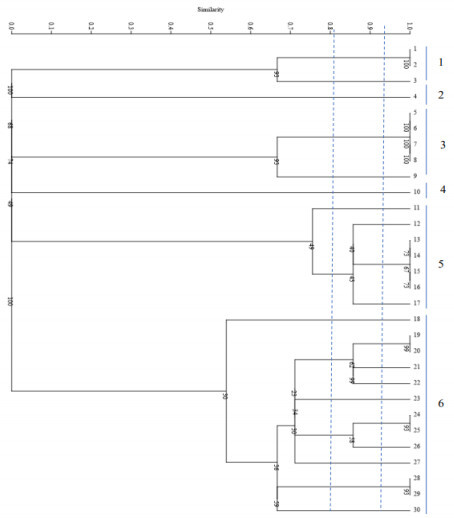
Phylogenetic tree based on the analysis of microsatellite results of *C. parapsilosis* samples

**Figure 6:**
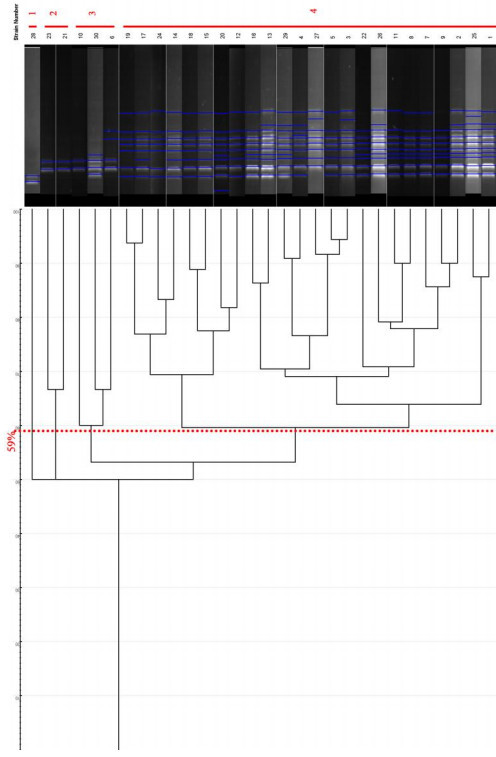
Dendrogram based on RAPD-PCR for *C. parapsilosis*

All strains of *C. parapsilosis* examined at a similarity level of 59% were divided into 4 distinct groups, such that 1 isolate was placed in the first group, 2 isolates in the second group, 3 isolates in the third group, and 24 isolates in the fourth group. The results of this part of the study are presented in Figure 5 (Fig. 5). The Simpson's coefficient calculated for RAPD-PCR was 35.0, indicating the low discriminatory power of this method for genotyping and polymorphism analysis of* C. parapsilosis* (Figure 6 (Fig. 6)).

Analysis of fifteen representative isolates revealed their positive reaction to various virulence factors, indicating their pathogenic potential. Remarkably, these isolates exhibited robust enzymatic activities, underscoring their high virulence. Additionally, the isolates demonstrated significant resistance against azole antifungal drugs

The amplification of DNA using three primers resulted in distinct multiband patterns ranging between 100 bp and 1,300 bp. Noteworthy is the observation of diverse DNA band sizes and electrophoretic patterns across different isolates, irrespective of their sources. These findings emphasize the effectiveness of the primers in identifying genetic variations within *C. albicans* spp., as evidenced by the polymorphic profiles generated (Figure 1 (Fig. 1), Figure 2 (Fig. 2), Figure 3 (Fig. 3)).

All fifteen* C. albicans* isolates exhibited unique RAPD profiles, indicating high genetic diversity. Eight isolates clustered together, while the remaining seven genotypes were represented by single isolates. The discrepancy observed in the RAPD profiles between isolates highlights genetic diversity within *Candida albicans *populations*. *Comparison of human and animal isolates revealed sequence homologies of 80-84%, indicating relatedness but not identical genetic backgrounds. Notably, no evidence of clonal relationship among the isolates was observed. 

These findings collectively provide valuable insights into the genetic diversity, pathogenicity, and antifungal resistance profiles of *Candida albicans* isolates, underscoring the importance of further molecular investigations to unravel the underlying mechanisms of virulence and drug resistance in this opportunistic pathogen.

The dendrogram (Figure 5 (Fig. 5)) illustrates the genetic similarity among *Candida albicans* isolates based on RAPD-PCR banding patterns. In our study, none of the *Candida albicans* isolates exhibited identical RAPD profiles when the results from all primers were aggregated to construct the dendrogram (see Figure 5 (Fig. 5)). The greatest genetic similarity was noted between a *Candida albicans* strain isolated from cow feces (A 27) and one obtained from a high vaginal swab (HVS 8b), showing an 84% homology. AB 87b, HVS 2a, A 43a, and AB 88b also displayed varying degrees of similarity among themselves and with A 27 and HVS 8b, albeit at different levels as evidenced by the distance of separation. Conversely, certain isolates (HVS 30, A 30, AB 81a, HVS16a, HVS 4b) appeared to exhibit significant genetic divergence from the remaining isolates, as indicated by the greater distance of separation. Therefore, it can be concluded that RAPD-PCR successfully delineated the genomic heterogeneity within the *Candida albicans* population.

## Discussion

The majority of isolates showed different banding patterns, indicating a high discriminatory power of the three primers used in genotyping [20]. Our RAPD analysis displayed a high level of heterogeneity, with most isolates showing less than 80% similarity. This suggests the presence of independent strains, consistent with previous studies that reported high levels of heterogeneity among human *Candida* isolates. Studies on *C. albicans* isolates from poultry birds in China suggested that these isolates were relatively independent but not completely separated from human isolates [21]. Previous research on the genetic relationship between human and animal isolates of *Candida albicans* found no evidence of species-specific lineages, although some degree of separation was observed [22].

According to the dendrogram generated in our study, the highest genetic similarity (84%) was observed between *Candida albicans* isolated from cow feces and a high vaginal swab, indicating a relatedness based on the dice coefficient. However, none of the isolates had identical RAPD profiles when all primers were combined. These findings are consistent with previous research on genetic homology in *Candida* species.

The primers effectively detected intraspecific polymorphism, demonstrating high discriminatory power in distinguishing *C. albicans* strains [23]. Previous studies have highlighted the importance of selecting primers that can effectively discriminate between *Candida* strains, as different primers may yield varying results [23]. Combining data from multiple primers can enhance discriminatory power in genotyping. RAPD analysis is a reliable, fast, and simple method that has been used extensively in studying pathogenic fungi [13]. It is useful in genotypic profiling, intra-specific identification, detecting small differences in nucleic acid content, assessing genetic relatedness, and determining transmission routes of fungi.

It was previously proposed by Pfaller et al. [3] that utilizing a combination of various molecular typing techniques could aid in distinguishing specific isolates from one another. Merseguel et al. [24] suggested that for insights into the natural history of hematogenous infection caused by the rapidly emerging *Candida* species, molecular studies targeting closely related and emerging species are suitable. Therefore, we argue that the use of molecular markers, such as RAPD-PCR, is highly beneficial in discerning the genetic diversity of *Candida* species. RAPD-PCR is a precise and easy to implement technique with numerous advantages. However, a significant drawback of RAPD-PCR is its limited interlaboratory reproducibility, necessitating highly standardized experimental procedures due to its sensitivity to reaction conditions. Variations in PCR conditions must be strictly standardized to avoid discrepancies in results. These limitations could impact the comparison of our results with those of other researchers investigating genetic variability among *Candida* species.

In conclusion, the dendrogram from our research displayed a notable separation of human and animal isolates. Some human and animal *C. albicans* isolates exhibited homologies between 80-84%, indicating a related but not identical relationship. Microevolutionary changes in a single isolate due to adaptation to changing environmental conditions and the possibility of unique strains in our dendrogram belonging to a different *Candida* species may have contributed to the variation in genotyping experiments [25]. The observed genetic variations could also be attributed to transmission barriers, given the differences in the exact locations of human and animal samples.

Our study further suggests that animals could serve as reservoirs for highly pathogenic and drug-resistant *Candida* species, posing a potential threat to humans. This is supported by the findings of Cordeiro et al. [26], who noted a high rate of resistance to azoles in *Candida* isolates from animals, along with expressed virulence factors that could pose a threat to human and animal health. Osman et al also highlighted the public health significance of bovines as potential sources of *C. albicans* zoonotic transmission to humans in urban-rural communities [27]. It is crucial to highlight that for strain transfer to occur between humans and animals, the recipient must have contact with a *C. albicans*-colonized animal or animal-derived products [18]. Based on our initial hypothesis and existing research, the transfer of Candida is considered from a human perspective, where animals serve as reservoirs for human *Candida* spp. This perspective is reasonable given the limited information on Candida isolation from animals and the focus on human candidiasis. Interactions with animals and their products are commonplace for most individuals, raising questions about the role of these animals in the spread of candidiasis. 

## Conclusions

In conclusion, it was found that RAPD-PCR is a suitable method for fingerprinting analysis, as it produced consistent fingerprints and clear banding patterns. The study demonstrated a significant separation between human and animal *Candida* isolates, with distinct genotypes identified. While no clonal relationship was established, the potential for transfer cannot be disregarded, especially in cases where humans come into contact with animals carrying virulent and resistant *Candida* strains. Future studies should incorporate larger sample sizes and more advanced molecular techniques, such as whole-genome sequencing, to further elucidate the epidemiological patterns and transmission dynamics of *Candida albicans*.

### Study Limitations

Although our sample size may not fully represent the genetic diversity within the study area, our findings on the genetic relatedness of *Candida* species align with previous studies. Furthermore, our isolates showed similarities in enzyme and antifungal patterns. Despite the high discriminatory power of RAPD-PCR, its inter-laboratory reproducibility remains a challenge, requiring validation with more robust molecular approaches such as MLST or WGS.

## Notes

### Competing interests

The authors declare that they have no competing interests.

### Authors’ ORCIDs

- Bayat M: https://orcid.org/0000-0001-8329-4283
- Yadegari MH: https://orcid.org/0000-0001-7976-3841
- Amini K: https://orcid.org/0000-0002-6419-3417

### Funding

None.

## Figures and Tables

**Table 1:**
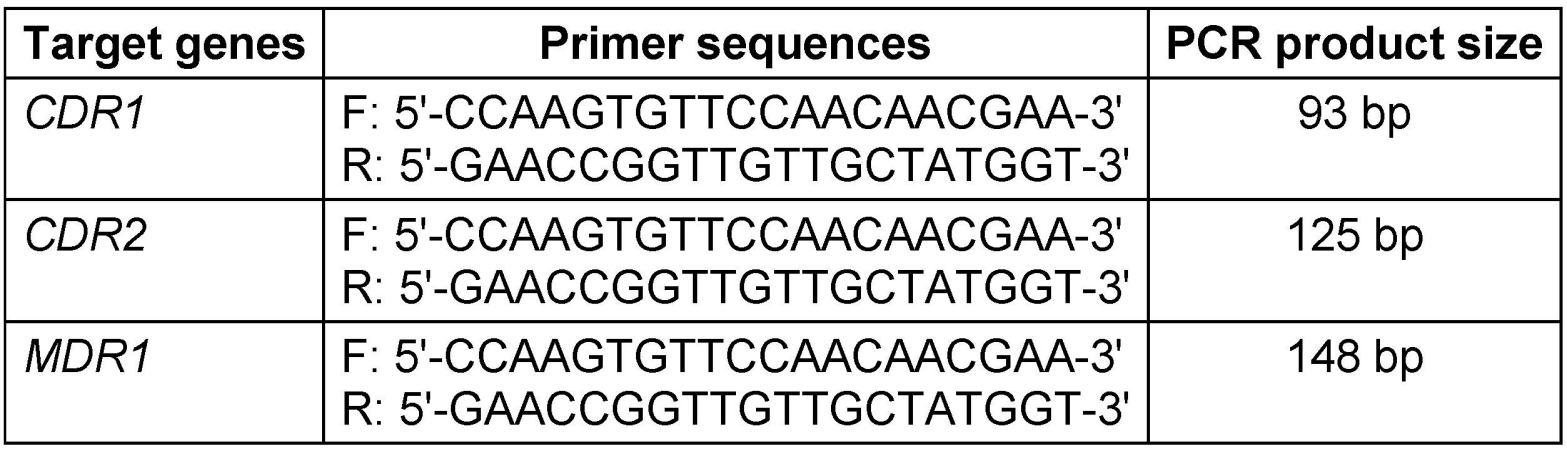
The primers used for antifungal resistance identification

**Table 2:**
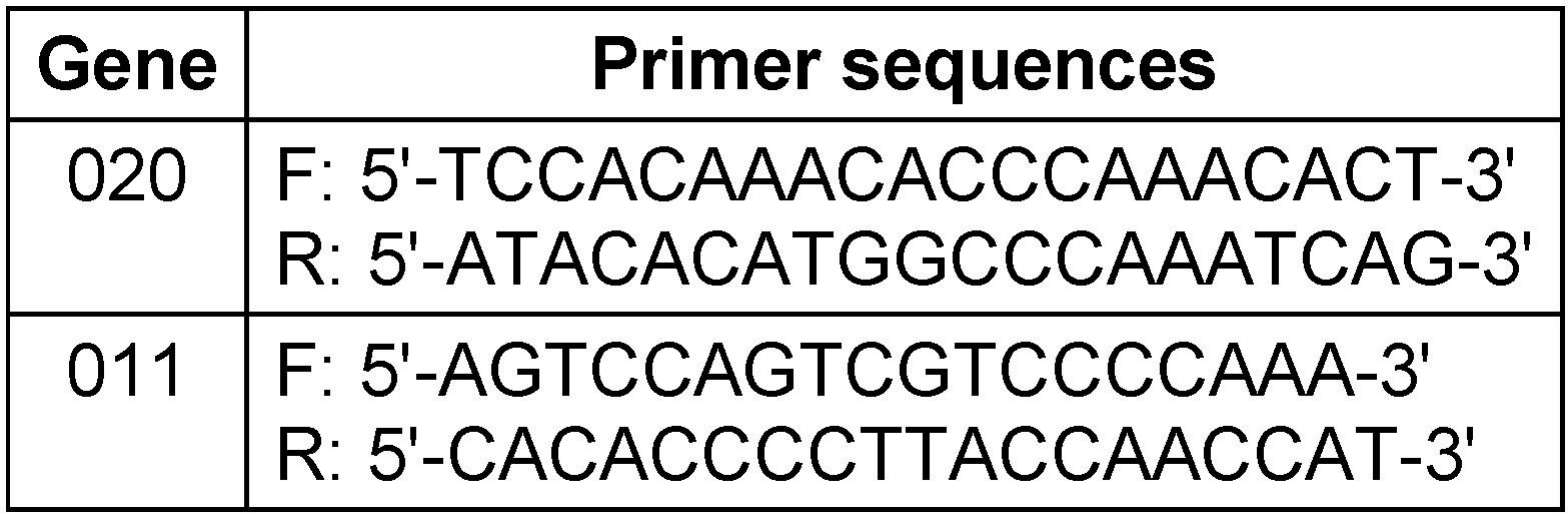
The primers used in the MLVA technique

**Table 3:**
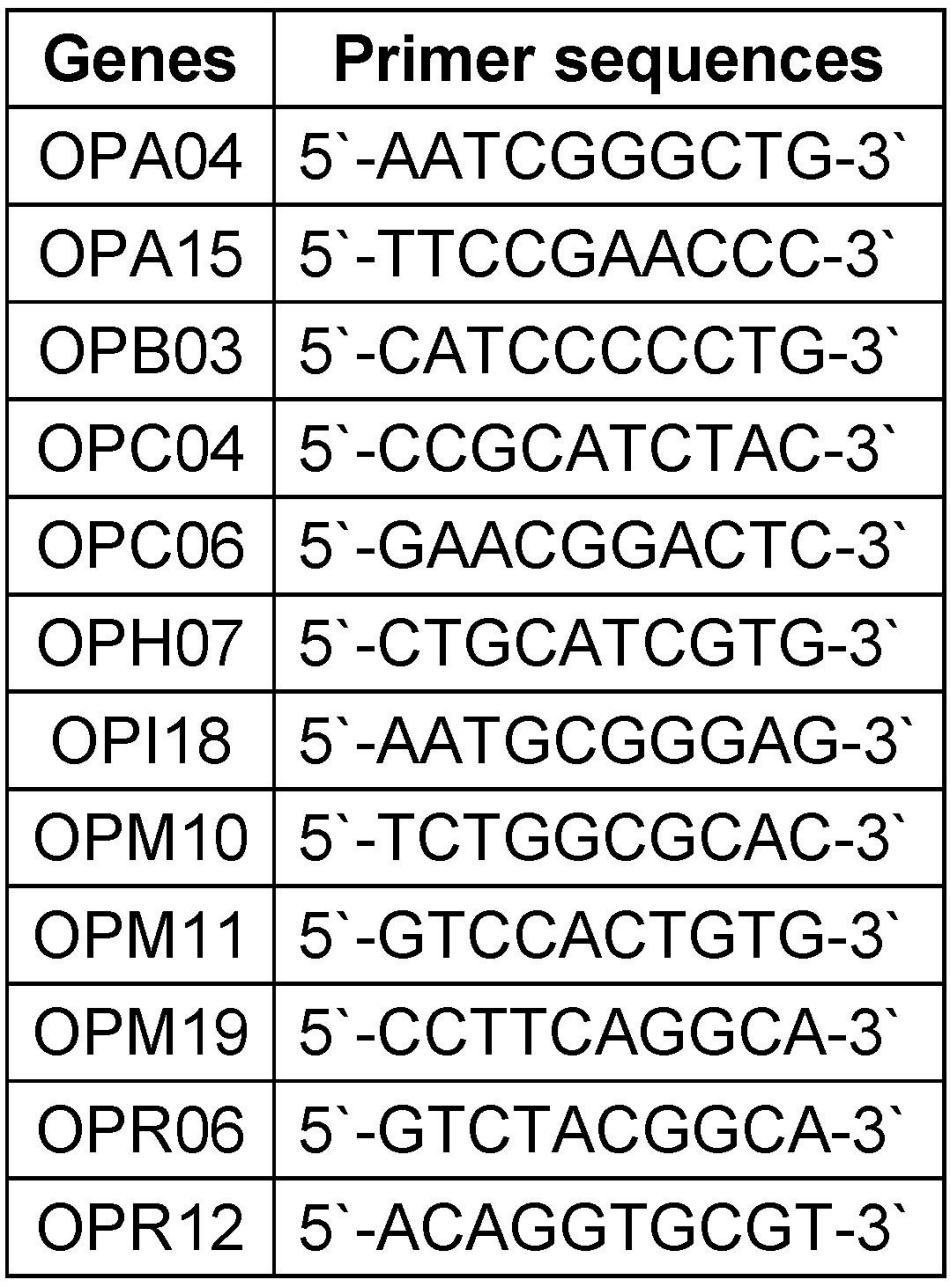
The primers used in the MLVA technique
